# Supplementary material for: Soil Fertilization with Palm Oil Mill Effluent Has a Short-Term Effect on the Bacterial Diversity of an Amazonian Agricultural Land Area
Source: Microorganisms. 2024 Mar 1;12(3):507. doi: 10.3390/microorganisms12030507 (PMC10972391; doi:10.3390/microorganisms12030507)
Supplement: Supplementary file 1 [file microorganisms-12-00507-s001.zip › microorganisms-2776674-supplementary.pdf]

Supplementary Material

Soil Fertilization with Palm Oil Mill Effluent Has a Short-Term Effect on the Bacterial Diversity of an Amazonian Agricultural Land Area

Johnes Pinto Sanches [1], Sávio Souza Costa [1], Diego Assis das Graças [1], Artur Silva [1], Guilherme Costa Baião [2], Rennan G. Moreira [3], Marcelo Murad Magalhães [4], Roberto Lisboa Cunha [4] and Rafael Azevedo Baraúna [1,*]

[1] Biological Engineering Laboratory, Innovation Space, Guamá Science and Technology Park, Belém 66075-750, PA, Brazil; johnes.sanches@icb.ufpa.br (J.P.S.); savscosta@gmail.com (S.S.C.); diego.a87@gmail.com (D.A.d.G.); arturluizdasilva@gmail.com (A.S.)

[2] Laboratory of Molecular and Computational Biology of Fungi, Department of Microbiology, Institute of Biological Sciences, Federal University of Minas Gerais, Belo Horizonte 30120-140, MG, Brazil; gcbaiao@gmail.com

[3] Multiuser Laboratories Center, Center of Genomics, Institute of Biological Sciences, Federal University of Minas Gerais, Belo Horizonte 31270-901, MG, Brazil; rennangm@gmail.com

[4] Brazilian Agricultural Research Corporation/EMBRAPA—Eastern Amazon, Belém 66095-100, PA, Brazil; marcelo.magalhaes@embrapa.br (M.M.M.); roberto.cunha@embrapa.br (R.L.C.)

* Correspondence: rabarauna@ufpa.br

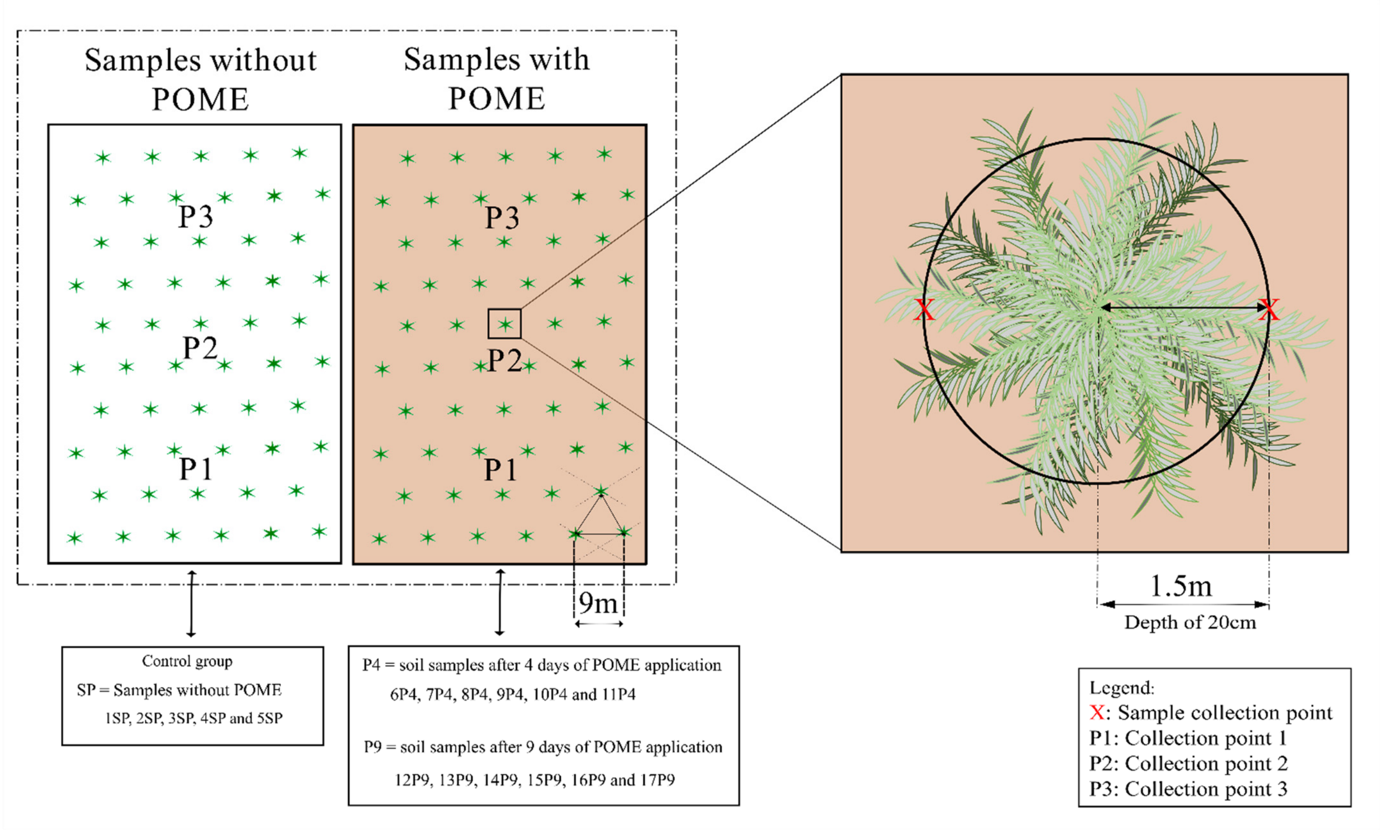

Figure S1. Sampling design.

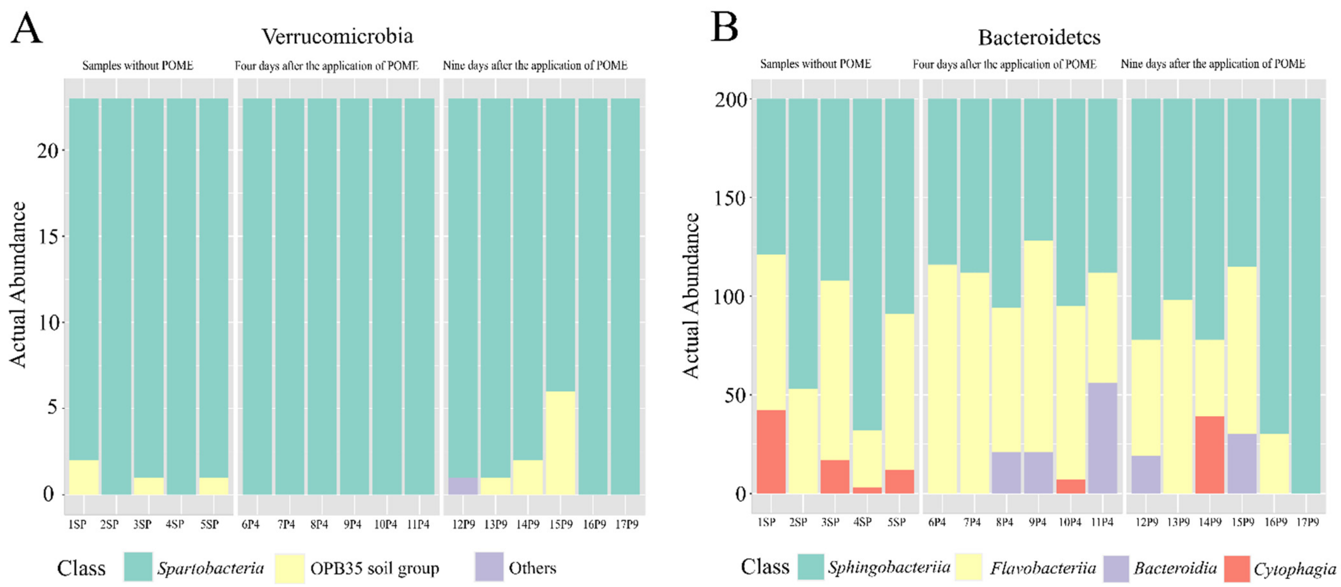

Figure S2. Histogram of relative species abundance for the phyla (A) Verrucomicrobia and (B) Bacteroidetes at the level of class.

Table S1. Sequencing metrics per sample.

Samples	DNA concentration (ng/uL)	Number of Reads (pairs)	Average Read Length	% GC	Illumina barcodes for primers	
					Index 1 I7	Index 2 I5
1SP	48.7	61278.0	251.0	54.0	CTAGTGCTCT	TGGAACAGTA
2SP	52.2	38656.0	251.0	49.0	GAAGCGGCAC	CGGCTCTACT
3SP	59.1	45212.0	251.0	52.0	TCCATTGCCG	GAATGCACGA
4SP	73.4	45455.0	251.0	49.0	GATCAAGGCA	CCTTGTTAAT
5SP	69.8	46719.0	251.0	50.0	GACTGAGTAG	GTTGATAGTG
6P4	63.6	50092.0	251.0	51.0	GAACTGAGCG	TCGTGGAGCG
7P4	68.8	63661.0	251.0	51.0	AGGTCAGATA	CTACAAGATA
8P4	84.7	66511.0	251.0	51.0	CGTCTCATAT	TATAGTAGCT
9P4	70.9	47626.0	251.0	51.0	ATTCCATAAG	TGCCTGGTGG
10P4	68.4	57342.0	251.0	53.0	AGTCAGACGA	ACCAGCGACA
11P4	84.5	62751.0	251.0	51.0	CGGTTACGGC	AAGACTATAG
12P9	92.5	68515.0	251.0	52.0	GACGAGATTA	ACATTATCCT
13P9	114.7	55642.0	251.0	52.0	AACATCGCGC	GTCCACTTGT
14P9	120.5	68692.0	251.0	55.0	GAGAATGGTT	TCGGCAGCAA
15P9	59.9	57483.0	251.0	51.0	CCGTATGTTC	CATACACTGT
16P9	173.1	53977.0	251.0	53.0	GAGTCATAGG	GTGTGGCGCT
17P9	116.6	59046.0	251.0	53.0	CTTGCCATTA	ATCACGAAGG

Table S2. Alpha-diversity values.

Samples	Number of sequence	ASV	Chao 1	Shannon	Simpson
1SP	22366	702	60.00	3.96	0.979
2SP	17235	516	54.00	3.85	0.977
3SP	20066	519	86.00	4.27	0.983
4SP	20524	587	67.00	4.05	0.981
5SP	21191	503	82.00	4.27	0.984
6P4	26878	262	158.25	4.52	0.984
7P4	28155	307	191.20	5.00	0.992
8P4	30970	575	241.00	5.26	0.994
9P4	21194	299	208.33	5.17	0.993
10P4	33397	342	204.39	4.49	0.981
11P4	32436	500	226.87	4.88	0.988
12P9	37435	513	222.33	4.95	0.991
13P9	29027	531	178.50	4.59	0.984
14P9	27978	766	138.00	4.73	0.989
15P9	30212	573	211.33	5.01	0.991
16P9	25285	634	186.33	5.03	0.992
17P9	25659	583	128.00	4.59	0.987
